# Laticifers are present in Acalyphoideae after all: new insights from leaf anatomy with implications for the systematics and evolution of Euphorbiaceae

**DOI:** 10.1093/aobpla/plaf006

**Published:** 2025-02-12

**Authors:** Clara Prandi Mouzella, Ana Angélica Sousa, Maria Beatriz Rossi Caruzo, Iris Montero-Muñoz, Renata Maria Strozi Alves Meira, Ricarda Riina

**Affiliations:** Universidade Federal de Viçosa, Departamento de Biologia Vegetal, Programa de Pós-Graduação em Botânica, Av. P.H. Rolfs, S/N, Campus Universitário, Viçosa, Minas Gerais 36570-000, Brazil; Universidade Federal de São Paulo - Unifesp, Instituto de Ciências Ambientais, Químicas e Farmacêuticas - ICAQF, Departamento de Ecologia e Biologia Evolutiva, Rua Prof. Artur Riedel, 257, Diadema, São Paulo 09972-270, Brazil; Universidade Federal de São Paulo - Unifesp, Instituto de Ciências Ambientais, Químicas e Farmacêuticas - ICAQF, Departamento de Ecologia e Biologia Evolutiva, Rua Prof. Artur Riedel, 257, Diadema, São Paulo 09972-270, Brazil; Real Jardín Botánico (RJB), CSIC, Plaza de Murillo 2, 28014 Madrid, Spain; Universidade Federal de Viçosa, Departamento de Biologia Vegetal, Programa de Pós-Graduação em Botânica, Av. P.H. Rolfs, S/N, Campus Universitário, Viçosa, Minas Gerais 36570-000, Brazil; Real Jardín Botánico (RJB), CSIC, Plaza de Murillo 2, 28014 Madrid, Spain

**Keywords:** anatomy, histochemistry, latex, ontogeny, secretory structures

## Abstract

Euphorbiaceae is among the main angiosperm families with a high number of laticiferous species. Although many of its species remain to be studied in terms of their anatomy, chemistry, and uses, there are some of recognized economic importance due to useful secondary compounds present in the latex. Acalyphoideae, one of the three major subfamilies, has traditionally been distinguished from the rest of Euphorbiaceae by the absence of latex and laticifers. To test this long-standing assumption, we anatomically analysed 40 species in 10 genera, representing six of the nine subclades of Acalyphoideae s.s., to examine the presence of laticifers using leaf blade and petiole sections. Laticifers were observed in all the studied species and consisted of multinucleate, elongated cells with dense cytoplasm. They were articulated and branched in *Acalypha*, *Bia*, and *Dalechampia*; this was further confirmed by ontogenetic analyses in *Acalypha accedens*, *A. brasiliensis*, and *A. poiretii*. Histochemical tests revealed lipids, proteins, mucilage, and starch in laticifers. Our results demonstrate that laticifers are present and common in Acalyphoideae and thus more widespread in Euphorbiaceae than previously known. The scarcity of detailed anatomical studies, and the often imperceptible latex exudation of most Acalyphoideae, are probably the main reasons that have misled field botanists and systematists in the past.

## Teaser

Our study shows that latex-secreting cells (laticifers) are widespread in the ecologically and economically important spurge family (Euphorbiaceae). Latex is a complex substance that plays key roles in plant ecology (e.g. defence against herbivores) and in benefits to human society (e.g. natural rubber, pharmaceuticals). Previous botanical knowledge pointed to the absence of laticifers as the main diagnostic feature of the subfamily Acalyphoideae, one of the three major groups of the spurge family. We analysed a representative sample of Acalyphoideae using methods of plant anatomy and found that laticifers were always present. As a result, the systematic characterization of Euphorbiaceae should be updated to consider laticifers as ubiquitous throughout the entire family.

## Introduction

Laticiferous plant species have been documented by observing the exudation of a milky fluid (latex) after a mechanical injury (Konno 2011) and by detailed anatomical studies (Lopes *et al.* 2009; Vitarelli *et al.* 2015; Pace *et al.* 2019; Pirolla-Souza *et al.* 2019; Medina *et al.* 2021). Latex is produced by laticifers, secretory structures composed of single or interconnected cells that form systems that permeate the entire body of the plant (Evert 2013). The presence of latex has been reported in several lineages across angiosperms, but Apocynaceae, Euphorbiaceae, Moraceae, and Papaveraceae stand out as the families with the highest number of latex-producing species (Metcalfe and Chalk 1983; Hagel *et al.* 2008; Konno 2011; Gama *et al.* 2017; Naidoo *et al.* 2020; Teixeira *et al.* 2020). In contrast, latex is rare in gymnosperms (known only in *Gnetum gnemon* L.) and in pteridophytes (reported so far in *Regnellidium diphyllum* Lindm.) (Metcalfe 1967). Such a wide range of phylogenetically unrelated groups reveals multiple independent origins of laticifers in vascular plants (Metcalfe and Chalk 1983; Agrawal and Konno 2009; Prado and Demarco 2018).

Plant biologists have used the presence of latex (and laticifers) as a key character in taxonomic and evolutionary studies (e.g. Demarco *et al.* 2006; Foisy *et al.* 2019; Pace *et al.* 2019; Medina *et al.* 2021). Latex production has been hypothesized to be the ancestral condition for Apocynaceae due to its presence in all the species of this family (Metcalfe 1967). Within Malpighiales, Vega *et al.* (2002) reported latex as a synapomorphy for the Galphimieae tribe (Malpighiaceae), providing further support to its monophyly. Later on, also in Malpighiaceae, Pace *et al.* (2019) expanded the presence of laticifers in lianoid lineages of this family. In Euphorbiaceae, the largest family of Malpighiales, laticifers have been reported to be common in Crotonoideae and Euphorbioideae, and lacking in Acalyphoideae (Webster 1975; Rudall 1987; Berry *et al.* 2005; Wurdack *et al.* 2005; Esser 2012; Secco *et al.* 2012; Horn *et al.* 2014). However, although these secretory structures have been considered absent in Acalyphoideae (Webster 1975; Rudall 1987; Wurdack *et al.* 2005), there have been a few scanty or poorly documented reports of laticifers in a few species (compiled in Rudall 1987; her table 2; Hayden and Hayden 2000; van Welzen *et al.* 2004; Norfaizal *et al.* 2012; Jangid and Gupta 2016).

Laticifers are classified as articulated or non-articulated (Metcalfe and Chalk 1989) and both types can be branched or unbranched (Fahn 1979; Rudall 1987; Mahlberg 1993; Wiedenhoeft *et al.* 2009; Pace *et al.* 2019). Articulated laticifers originate from several cells, which may or not undergo lateral anastomosis with adjacent rows of laticifers. Non-articulated laticifers develop from a single cell that elongates as the plant grows, by sequential mitotic divisions without cytokinesis, forming a multinucleate cell which may or may not branch (Rudall 1987; Hagel *et al.* 2008; Wiedenhoeft *et al.* 2009). When laticifers are broken by mechanical injury, their entire protoplasm overflows from the wounded tissue, including the latex stored inside the vacuoles (Demarco *et al.* 2006; Prado and Demarco 2018). Overall, the anatomy of laticifers is similar across different plant lineages, but the chemical composition of the latex can vary even among closely related species (Ramos *et al.* 2020).

Latex is an emulsion composed of particles (such as organic acids, salts, proteins, polysaccharides, mucilage, starch grains, lipids, fatty acids, fats, sterols, rubber, alkaloids, and phenolic compounds) mixed in an aqueous fluid (Fahn 1979; Agrawal and Konno 2009). It can be translucent (colourless), white (milky), reddish, yellowish, greenish, or orange, and is known to act as a defence against herbivores and pathogens (Fahn 1979; Konno 2011; Castelblanque *et al.* 2016; Salomé-Abarca *et al.* 2021; Huber 2024). The latex of several plant families, including some Euphorbiaceae, is of economic importance due to its use in the production of commercial goods such as rubber from *Hevea brasiliensis* (Willd. ex A.Juss.) Müll.Arg., and other useful compounds exploited from several *Croton* and *Euphorbia* species (Riina *et al.* 2009; Mwine and Van Damme 2011; King *et al.* 2020).

The present study aims to fill a long-standing gap in the anatomical knowledge of the family Euphorbiaceae, namely whether laticifers are present and widespread in Acalyphoideae as they are in Crotonoideae and Euphorbioideae. We will address this gap by exploring the leaf anatomy of several genera spanning the major lineages of Acalyphoideae to verify the occurrence of laticifers, as well as to describe their morphology, ontogeny, histochemistry, and distribution patterns across taxonomic groups.

## Materials and methods

**Figure 1.**
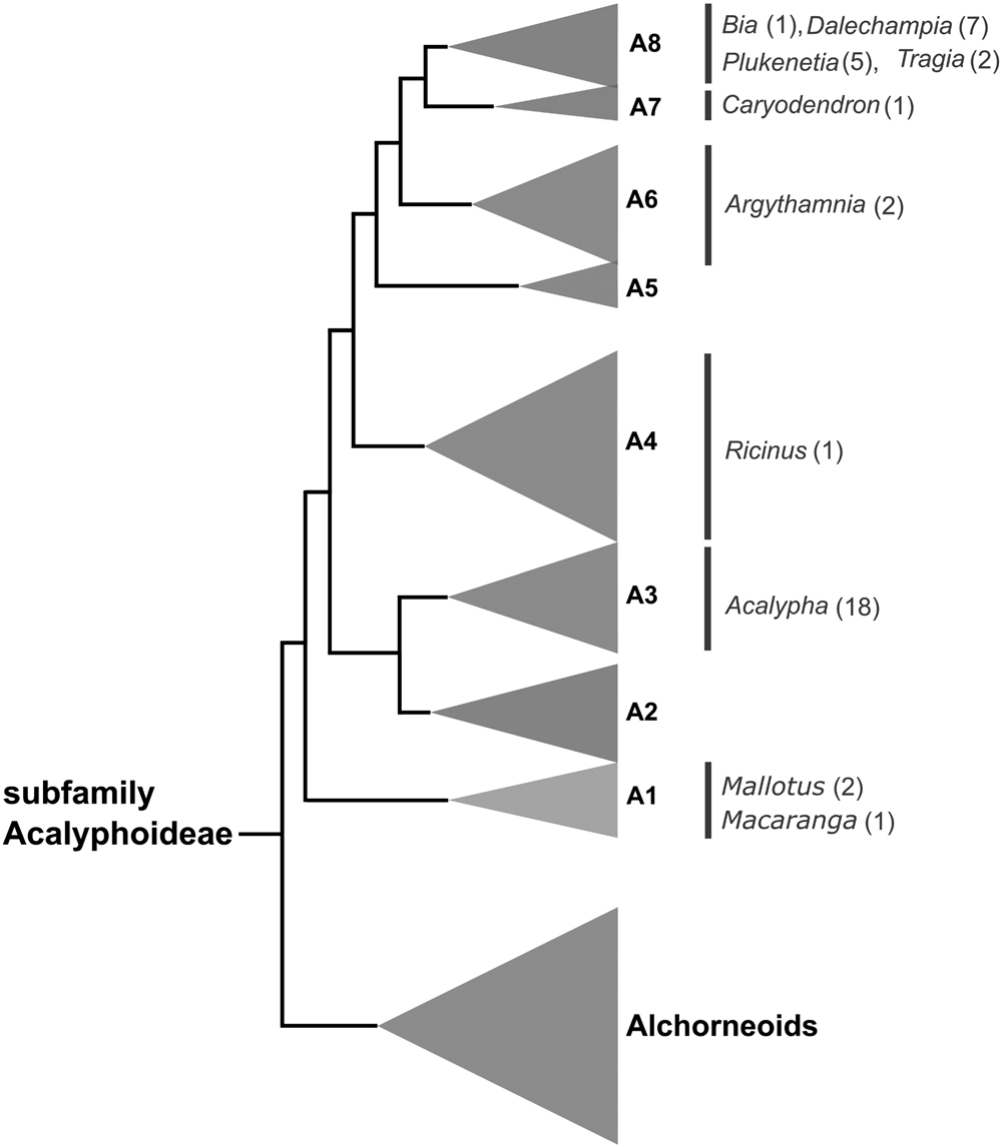
Schematic cladogram showing the sampling of genera across the major clades of Acalyphoideae s.s. used in this study. The number of sampled species per genus is indicated in parentheses. Specimens from clades A2, A5, and ‘Alchorneoids’ were not available for this study. Cladogram and clade labels follow Wurdack *et al.* (2005).

Taxon sampling was carried out following the phylogenetic framework of Wurdack *et al.* (2005). We selected different genera representing six of the nine clades of subfamily Acalyphoideae s.s. (Fig. 1). In most cases, we analysed multiple specimens per species when available. Unfortunately, we could not obtain suitable tissue material from the ‘Alchorneoids’, A2, and A5 clades on time for this study (Fig. 1).

Information about taxa, taxonomic authorities, voucher information, herbarium specimen barcode, and locality of the analysed specimens is included in Supplementary Appendix 1. Available data about exudation and its colour, plant habit, growth form, and geographic distribution were obtained from several online and literature sources (Whitmore 1981; Romero and Sanguinetti 1989; Forster 1999; Flora do Brasil 2020), as well from the labels of herbarium specimens. We evaluated 40 species that belong to 10 genera and six clades of Acalyphoideae s.s. (Supplementary Appendix 1, Fig. 1): *Acalypha* (18 spp.), *Argythamnia* (2 spp.), *Bia* (1 sp.), *Caryodendron* (1 sp.), *Dalechampia* (7 spp.), *Macaranga* (1 sp.), *Mallotus* (2 spp.), *Plukenetia* (5 spp.), *Ricinus* (1 sp.), and *Tragia* (2 spp.). Below we provide general relevant information about each of the sampled genera; approximate number of species is based on current taxonomic knowledge (e.g. World Flora Online Plant list, https://wfoplantlist.org/).

*Acalypha* is the third species-rich genus of Euphorbiaceae with about 500 species distributed in tropics and subtropics worldwide (Cardiel *et al.* 2023; Montero-Muñoz *et al.* 2023). Species vary from herbs to sprawling shrubs and small trees occupying a wide diversity of habitats from tropical rain forests to subdesertic areas. *Argythamnia* has 68 species of herbs, shrubs, or subshrubs, native to dry habitats of the New World (Külkamp *et al.* 2023). *Bia* consists of five species of climbing herbs, endemic to the Neotropics. *Caryodendron* includes four tree species endemic to the Neotropics and distributed in humid forests. *Dalechampia* has about 118 species of shrubs to subshrubs, herbs, or vines, distributed in several tropical biomes. *Macaranga* has about 280 species distributed mainly on humid forests of the Paleotropics (Slik and van Welzen 2001). *Mallotus* comprises around 112 species of shrubs and small trees found in tropical areas of Africa, Asia, and Oceania (Australia). *Plukenetia* comprises 26 shrubby or lianescent species distributed in the Neotropics, mainland Africa, and Madagascar. The monotypic *Ricinus* (*R. communis* L.) is a shrub or small tree introduced in many countries outside its native area (east tropical Africa). Finally, *Tragia* includes herbaceous climbing species (c. 150 spp.) widely distributed in America, Africa, India, and Australia (Cardinal-McTeague and Gillespie 2016).

Samples of fully expanded leaves were taken from herborized materials deposited in herbaria VIC, BHCB, SP, SPF, and EAC (acronyms follow Thiers 2024, continuously updated). Information about geographic locality and field observations contained on the labels of each specimen was recorded. The taxonomic identity of all specimens was confirmed or revised by the taxonomists in our research team (all working on Euphorbiaceae). After analysis under a stereoscopic microscope (Olympus 110AL2X, Tokyo), the samples were submitted to the reversion process, dehydrated in an ethylic series, and stored in 70% ethanol (Smith and Smith 1942).

We also conducted fieldwork to obtain and fix fresh leaf samples for the identification of compounds present in the leaf tissue. We collected fresh material from 11 species: *Acalypha accedens* Müll.Arg., *A. alopecuroidea* Jacq., *A. amblyodonta* Müll.Arg., *A. brasiliensis* Müll.Arg., *A. herzogiana* Pax & K.Hoffm., *A. hispida* Burm.f., *A. wilkesiana* Müll.Arg., *A. poiretii* Spreng., *Bia alienata* Didr., *Dalechampia* sp. 1, and *Ricinus communis* (Supplementary Appendix 1). The presence and colour of any exudate were recorded after cuttings for sampling in the field. To avoid extravasation of exudate during field sampling, heated razor blades were used. Fresh field samples were immediately fixed and stored in neutral buffered formalin (Kraus and Arduin 1997). Transversal and longitudinal sections, obtained using a table microtome (LPC, Rolemberg and Bhering Trade and Import, Belo Horizonte, Brazil), were histochemically tested. Shoot apex were also sampled from *A. accedens*, *A. brasiliensis*, and *A. poiretii* for ontogenetic analysis.

For anatomical characterization, samples from herborized material (stored in 70% ethanol) and fixed samples from the field were dehydrated in an ethanol series (70%, 80%, 90%) and embedded in methacrylate (Historesina Leica Microsystems Nussloch GmbH, Heidelberg, Germany). Transversal and longitudinal sections of 5 μm in thickness were obtained, using an automatic rotary microtome (model RM2155, Leica Microsystems Inc., Deerfield, IL, USA), from the petiole, the middle third of the leaf blade (midrib and mesophyll), and the shoot apex. The sections were stained with toluidine blue at pH 4.4 (O’Brien and McCully 1981) and the slides were mounted with synthetic resin (Permount, Fisher Scientific, Fair Lawn, NJ, USA).

Histochemical tests were applied to samples sectioned on an automatic rotary microtome and to those sectioned on a table microtome (LPC-Spencer). The following reagents were used: periodic acid and reagent of Schiff (PAS) for total polysaccharides (McManus 1948), Ruthenium Red for pectic and mucilage compounds (Johansen 1940), Lugol reagent for starch (Johansen 1940), Ponceau’s Xylidine for proteins (Vidal 1970), and Oil red for lipids (Pearse 1968).

Observations and photographic documentation were performed using a light microscope (AX70TRF, Olympus Optical, Japan) equipped with an image capture system (Ax Cam, Zeiss, Germany) in the Laboratory of Plant Anatomy of the Federal University of Viçosa, Minas Gerais, Brazil.

## Results

**Figure 2.**
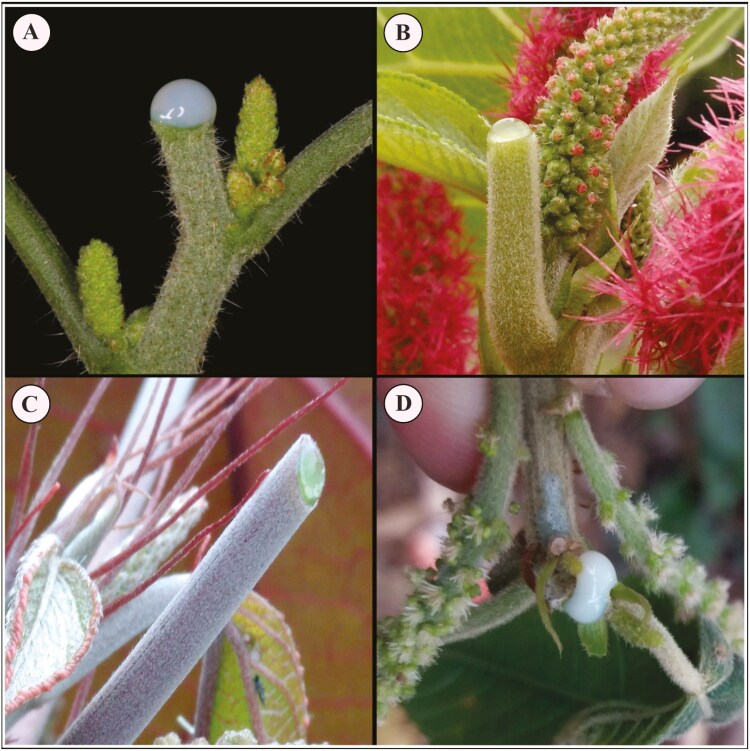
Milky or translucent latex-like secretion observed in the field in four *Acalypha* species: *A. brasiliensis* (A), *A. hispida* (B), *A. wilkesiana* (C), and *A. macrostachya* (D). Photos: Ana Angélica Sousa (A); Clara Prandi Mouzella (B, C); Otávio Luis Marques da Silva (D).

We found laticifers in the histological sections of all analysed genera (10), species (40), and specimens (72) (Table 1), regardless of their growth form, habitat, distribution, or clade to which they belong. Of the 11 species (four genera) sampled from fresh field-collected material (Supplementary Appendix 1), a latex-like secretion was observed in only five species of genus *Acalypha: A. accedens*, *A. amblyodonta*, *A. brasiliensis* (Fig. 2A), *A. hispida* (Fig. 2B), and *A. wikkesiana* (Fig. 2C). Latex was also reported in *A. macrostachya* Jacq. (Fig. 2D), a species analysed here using herbarium material (field image provided by collector). The secretion from the cuttings made on the stems and petioles of these species varied from a translucent to milky liquid depending on the species (Fig. 2). In contrast, *A. alopecuroidea*, and *A. herzogiana* did not show any evident latex, nor did the *Bia*, *Dalechampia*, and *Ricinus* specimens sampled in the field.

**Table 1.** Summary of the observations on laticifers and their main characteristics resulting from the analysis of leaf sections (blade and petiole) of 72 specimens representing 40 species and 10 genera of Acalyphoideae s.s.

Taxon name	Voucher	Laticifers					Histochemical tests			
		Presence	Type	Branching	Assoc. to phloem	In cortex	Mucilage	Protein granules	Starch grains	Lipid particles
*Acalypha accedens*	SP279979	+	A	H	+	+	0	0	0	0
*A. accedens*	SP489614	+	A	H	+	+	0	0	0	0
*A. accedens*	BHCB37233	+	A	H	+	+	0	0	0	0
*A. accedens*	*VIC53539	+	A	H	+	+	+	0	−	+
*A. amblyodonta*	BHCB26682	+	A	−	+	+	0	0	0	0
*A. amblyodonta*	*VIC53538	+	A	−	+	+	−	−	−	0
*A. alopecuroidea*	*VIC53541	+	A	−	+	+	−	+	−	0
*A. brasiliensis*	SP488255	+	A	−	+	+	0	0	0	0
*A. brasiliensis*	SP489606	+	A	−	+	+	0	0	0	0
*A. brasiliensis*	*VIC53547	+	A	−	+	+	−	+	+	−
*A. brasiliensis*	SPF99505	+	A	−	+	+	0	0	0	0
*A. brasiliensis*	SP75942	+	A	−	+	+	0	0	0	0
*A. brasiliensis*	SP357837	+	A	−	+	+	0	0	0	0
*A. brasiliensis*	ESA87524	+	A	−	+	+	0	0	0	0
*A. brasiliensis*	*SP489617	+	A	−	+	+	0	0	0	0
*A. brasiliensis*	SP35517	+	A	−	+	+	0	0	0	0
*A. brasiliensis*	BHCB6323	+	A	−	+	+	0	0	0	0
*A. communis*	BHCB63235	+	A	−	+	+	0	0	0	0
*A. communis*	VIC55844	+	A	−	+	+	0	0	0	0
*A. digynostachya*	BHCB61418	+	A	−	+	+	0	0	0	0
*A. diversifolia*	BHCB139376	+	A	Y	+	+	0	0	0	0
*A. diversifolia*	VIC23893	+	A	Y	+	+	0	0	0	0
*A. gracilis*	BHCB76400	+	A	Y	+	+	0	0	0	0
*A. gracilis*	SP489608	+	A	Y	+	+	0	0	0	0
*A. herzogiana*	*VIC53545	+	A	−	+	+	−	+	−	−
*A. herzogiana*	VIC22534	+	A	−	+	+	0	0	0	0
*A. hispida*	*VIC053823	+	A	−	+	+	−	+	−	−
*A. klotzschii*	BHCB11304	+	A	−	+	+	0	0	0	0
*A. macrostachya*	VIC856	+	A	−	+	+	0	0	0	0
*A. macrostachya*	INPA203328	+	A	−	+	+	0	0	0	0
*A. multicaulis*	EAC33254	+	A	Y	+	+	0	0	0	0
*A. multicaulis*	EAC32472	+	A	Y	+	+	0	0	0	0
*A. peckoltii*	SP312879	+	A	−	+	+	0	0	0	0
*A. poiretii*	EAC55830	+	A	Y	+	+	0	0	0	0
*A. poiretii*	*SP48965	+	A	Y	+	+	0	0	0	0
*A. velamea*	BHCB9141	+	A	−	+	+	0	0	0	0
*A. velamea*	SP167417	+	A	−	+	+	0	0	0	0
*A. velamea*	SP384372	+	A	−	+	+	0	0	0	0
*A. villosa*	HUU34451	+	A	−	+	+	0	0	0	0
*A. villosa*	EAC40795	+	A	−	+	+	0	0	0	0
*A. villosa*	EAC4216	+	A	−	+	+	0	0	0	0
*A. villosa*	EAC16112	+	A	−	+	+	0	0	0	0
*A. villosa*	EAC8563	+	A	−	+	+	0	0	0	0
*A. wilkesiana*	*VIC53546	+	A	−	+	+	−	+	−	−
*A. wilkesiana*	ESA87524	+	A	−	+	+	0	0	0	0
*Argythamnia fasciculata*	HUEFS137261	+	−	Y	+	−	0	0	0	0
*Argythamnia* sp.	HUEFS137934	+	−	−	+	−	0	0	0	0
*Bia alienata*	*VIC53544	+	A	−	+	+	−	+	−	−
*Caryodendron janeirense*	ESA120112	+	−	−	+	−	0	0	0	0
*Dalechampia adscendens*	SPF225969	+	A	−	+	−	0	0	0	0
*D. ficifolia*	VIC26804	+	A	−	+	−	0	0	0	0
*D. humilis*	SP442645	+	A	−	+	−	0	0	0	0
*D. pentaphylla*	VIC5283	+	A	−	+	−	0	0	0	0
*D. triphylla*	VIC7817	+	A	−	+	−	0	0	0	0
*Dalechampia* sp. 1	*VIC53542	+	A	−	+	+	−	−	−	−
*Dalechampia* sp. 2	SPF146013	+	A	−	+	−	0	0	0	0
*Macaranga heudelotti*	SP83179	+	−	Y	+	−	0	0	0	0
*Mallotus claoxyloides*	SP246983	+	−	−	+	−	0	0	0	0
*M. nesophilus*	SP226804	+	−	−	+	+	0	0	0	0
*Plukenetia brachybotrya*	INPA111455	+	−	−	+	−	0	0	0	0
*P. brachybotrya*	INPA229921	+	−	−	+	−	0	0	0	0
*P. loretensis*	SP444649	+	−	−	+	−	0	0	0	0
*P. loretensis*	INPA177605	+	−	−	+	−	0	0	0	0
*P. multiglandulosa*	INPA36048	+	−	−	+	−	0	0	0	0
*P. serrata*	SP475924	+	−	−	+	−	0	0	0	0
*P. serrata*	SP262251	+	−	−	+	−	0	0	0	0
*P. serrata*	SP367632	+	−	−	+	−	0	0	0	0
*P. volubilis*	SP292378	+	−	−	+	−	0	0	0	0
*P. volubilis*	INPA115525	+	−	−	+	−	0	0	0	0
*Ricinus communis*	*VIC53543	+	−	Y	+	+	−	+	−	−
*Tragia incana*	SP51683	+	−	−	+	−	0	0	0	0
*Tragia* sp.	S 51683	+	−	−	+	−	0	0	0	0

Anatomical sections were obtained from fresh field samples (11 specimens*) and from herbarium material (61 specimens). Codes: A = articulated laticifer; H = H-shaped branch; Y = Y-shaped branch; + = observed; − = not observed; 0 = not analysed.

**Figure 3.**
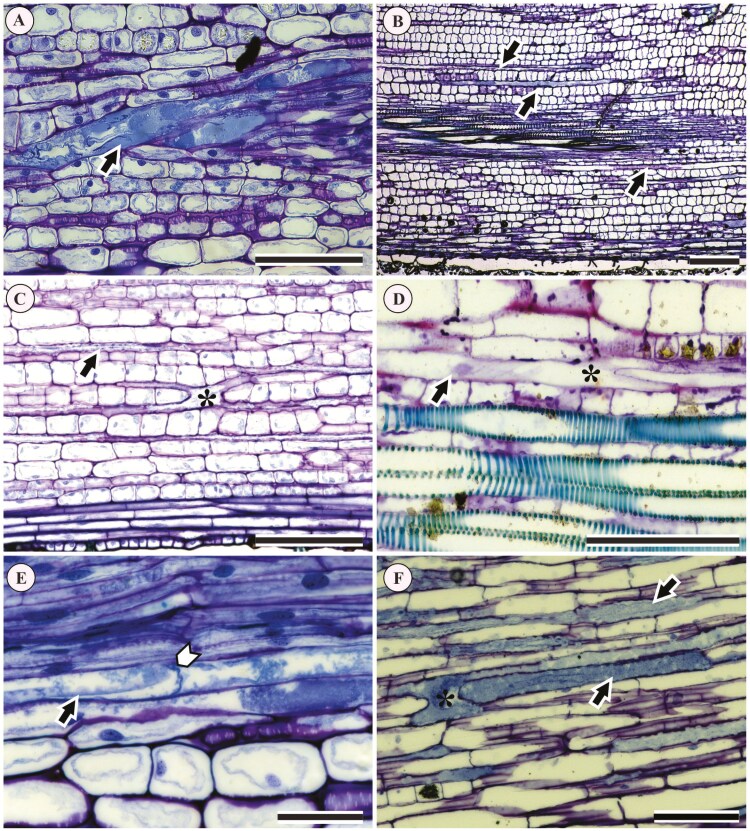
Laticifers in Acalyphoideae species visualized in longitudinal sections of the petiole stained with Toluidine blue. (A, B) Elongated laticifers (arrows); (A, F) Laticifers with dense cytoplasm and a granular appearance (arrows); (A–D) Nuclei evident in laticifers; (C, D) Branches of laticifers. (A) *Acalypha accedens*; (B) *Acalypha brasiliensis*; (C, D) *Acalypha wilkesiana*; (E) *Bia alienata*; (F) *Dalechampia* sp. 1. Black arrows indicate laticifer cells; white arrows indicate transversal walls; asterisks indicate Y-shaped branching in a laticifer. Scales: (A, D–F) 50 μm; (B) 200 μm.

In the leaf blade sections, it was challenging to distinguish laticifers from the other cell types adjacent to them because laticifers were often empty (i.e. the dense cytoplasm of a laticifer cannot be stained when devoid of content) due to latex leakage during both collection and specimen preparation. However, longitudinal and transverse sections of the petiole showed more evident content inside the laticifer (Fig. 3A–F), especially in those located in the vascular system (Fig. 3). For this reason, our reports (Table 1) and descriptions of laticifers below are mostly based on analysis of petiole sections, with the exception of the *Plukenetia* specimens that yielded observations from both blade and petiole sections.

**Figure 4.**
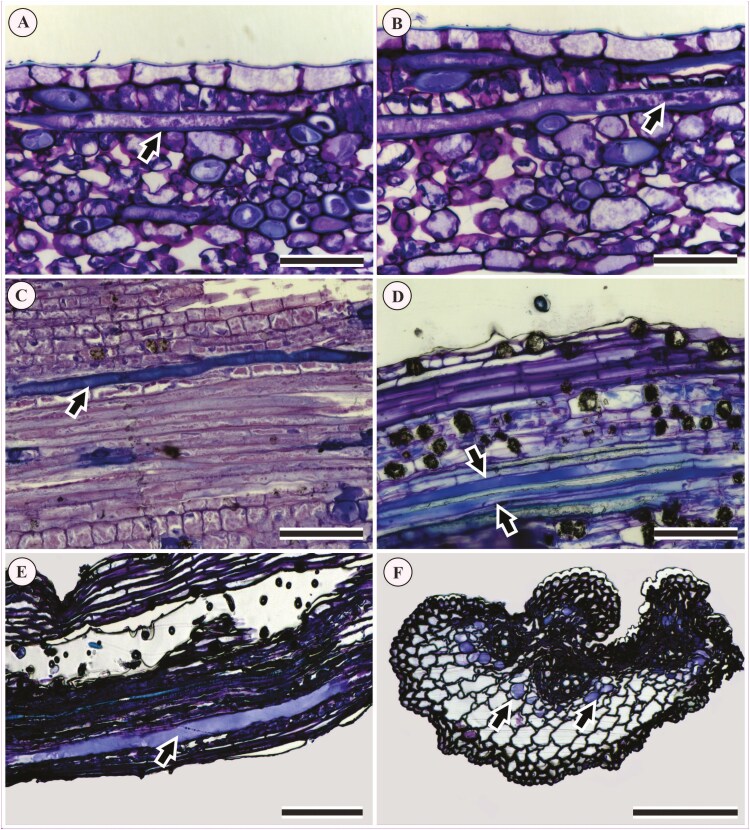
Laticifers in Acalyphoideae species as visualized in longitudinal and transverse sections of the leaf blade and petiole, stained with Toluidine blue. (A, B) Elongated laticifers in cross-section of the leaf blade showing several nuclei per cell (*Plukenetia loretensis*); (C–E) Laticifers in longitudinal section of the petiole: (C) *Acalypha diversifolia*; (D) *A. herzogiana*; (E) *A. villosa*; (F) Laticifers in cross-section of the petiole of *A. villosa*. Arrows indicate the laticifers and their nuclei. Scales: (A, B) 50 μm; (C, D) 100 μm; (E, F) 200 μm.

Laticifers formed a continuous tube made up of elongated cells, strongly stained blue (Fig. 3A, C, and F), achlorophyllous, multinucleate (Fig. 3E and F), with thin walls, and with a dense and granular cytoplasm (Fig. 3A, C, and D). In some species, laticifers showed H-shaped branches, as in *A. accedens* (Fig. 3A) and *A. brasiliensis* (Fig. 3B). On the other hand, laticifers had Y-shaped branches in *B. alienata* (Fig. 3E) as well as in *A. brasiliensis, A. diversifolia* Jacq., *A. gracilis* Spreng., *A. multicaulis* Müll.Arg., *A. poiretii*, *Argythamnia fasciculata* (Vahl ex A.Juss.) Müll.Arg., and *R. communis* (not shown). Branched laticifers were not observed in the rest of the sampled species/genera (Table 1). In general, laticifers were easily distinguished from the adjacent cells (Fig. 4B, C, E, and F), which had an isodiametric shape, and contained a large vacuole, and numerous chloroplasts (Fig. 3A–F). The only observations from leaf blade sections were obtained from *Plukenetia* samples, which presented elongated laticifers with prominent nuclei (Fig. 4A and B).

**Figure 5.**
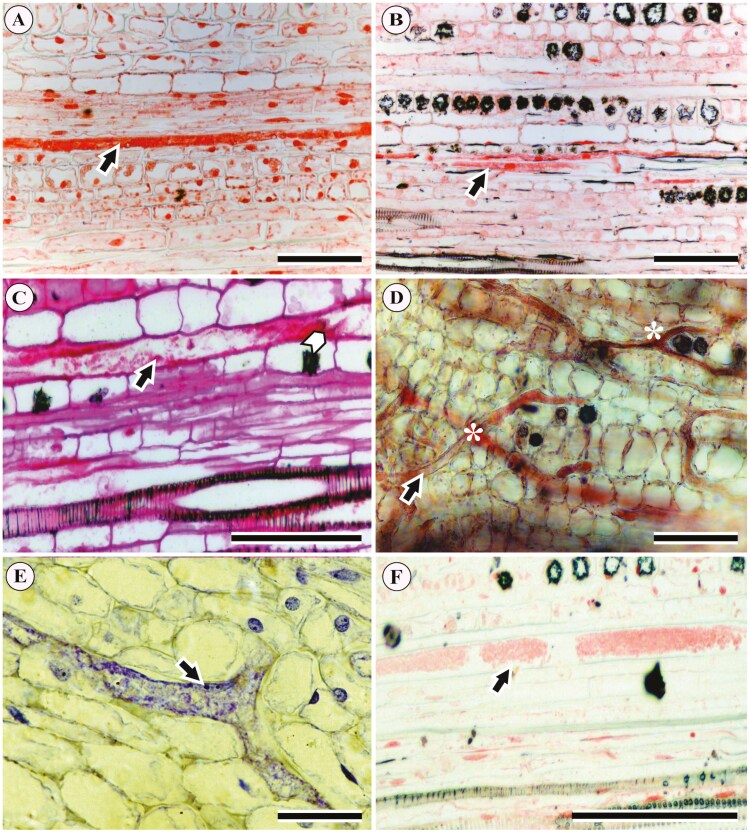
Results of histochemical tests showing the content of laticifers in longitudinal sections of the petiole in Acalyphoideae species: (A) protein granules in *Acalypha wilkesiana*; (B) protein granules in *Ricinus communis*; (C) mucilage and an intact transverse wall in *Bia alienata*; (D) lipid particles in *Acalypha accedens*; (E) starch grains in *Acalypha brasiliensis*; (F) protein granules in *Acalypha hispida*. Black arrows indicate laticifer cells; the white arrow indicates a transversal wall in a laticifer; asterisks indicate Y-shaped branching in a laticifer. Scales: 100 μm.

Laticifers were predominantly associated with the phloem in all taxa evaluated, as shown in transverse and longitudinal planes for several *Acalypha* species (Fig. 4E and F). Laticifers were also visualized in the cortex in several taxa, including all *Acalypha* species and some species from other genera, such as *B. alienata*, *Dalechampia* sp. 1 (e.g. Figs 3A–C and 4C and D), *Mallotus nesophilus* Müll.Arg. and *R. communis* (not shown). In transverse sections, laticifers associated with the vascular bundles were present as cells with an irregular outline, dense cytoplasm, and strongly stained blue, as observed in several species including *Acalypha villosa* Jacq. (Fig. 4F), *A. alopecuroidea*, *R. communis*, *D. humilis* Müll.Arg., and *M. nesophilus* (not shown). Transverse walls, indicating the presence of articulated laticifers, were observed in all the sampled species of *Acalypha*, *Bia*, and *Dalechampia* (Table 1), as shown in *A. accedens* (Fig. 3A), and in *B. alienata* (Fig. 5C). Regarding the presence of non-articulated laticifers, we did not observe this type in any of the analysed samples.

The histochemical tests on the cytoplasm of the laticifers (Fig. 5) revealed the following features (Table 1). Protein granules (Xylidine Ponceau) were observed in *Acalypha hispida* (Fig. 5F), *A. wilkesiana* (Fig. 5A), and *R. communis* (Fig. 5B), also in *A. accedens*, *A. alopecuroidea*, *A. amblyodonta*, *A. brasiliensis* (not shown). Lipid particles (Oil red) were found in *A. accedens* (Fig. 5D). Rod-shaped starch grains (Lugol) were observed in *A. brasiliensis* (Fig. 5E). Mucilage and pectin (Ruthenium Red) were documented in *B. alienata* (Fig. 5C) and *A. accedens* (not shown; Table 1). These tests also showed the presence of transverse walls in *B. alienata* (Fig. 5C) and Y-shaped branches in the laticifers of *A. accedens* (Fig. 5D). Finally, we observed spherical starch grains in laticifers and their surrounding parenchyma cells only in *A. brasiliensis* (Fig. 5E; Table 1).

**Figure 6.**
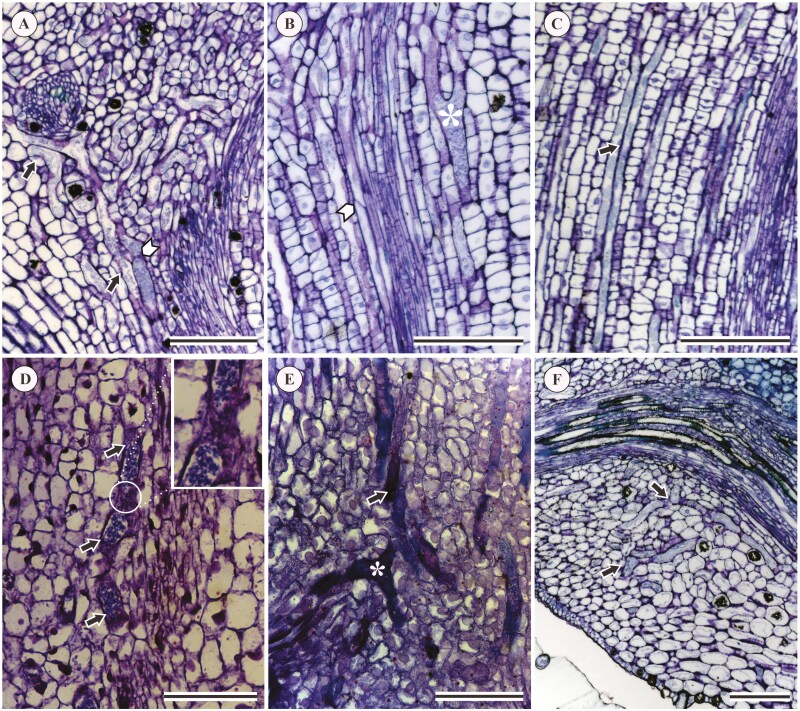
Results of ontogenetic analysis showing longitudinal sections of shoot apex in *Acalypha* species: (A, B) *A. accedens*; (C–E) *A. brasiliensis*; (F) *A. poiretii*. Black arrows indicate laticifer cells; white arrows indicate transversal walls; asterisks indicate Y-shaped branching in a laticifer. Scales: (A–C, F) 200 μm; (D, E) 150 μm.

The ontogenetic analyses showed articulated laticifers with ramifications and transverse walls in *A. accedens* (Fig. 6A and B), *A. brasiliensis* (Fig. 6C–E), and *A. poiretii* (Fig. 6F) (Table 1). They also revealed that elongated, multinucleated, thin-walled laticifers (Fig. 6A and B) with dense and granular cytoplasm (Fig. 6A–F) were located between the procambial and the fundamental meristem cells of the cortex and medulla. These results allowed us to confirm without doubt the presence of articulated laticifers in the leaves of *Acalypha* (Table 1).

## Discussion

Our study is the first to conduct a broad taxon sampling, guided by a molecular phylogenetic framework, for an anatomical survey of laticifers in the subfamily Acalyphoideae (Euphorbiaceae). Of the nine main clades of Acalyphoideae s.s. recovered by Wurdack *et al.* (2005), we detected the presence of laticifers in six clades represented by 10 genera and 40 species. Our findings suggest that these specialized secretory cells are probably widespread across Acalyphoideae, as they are in the rest of the family Euphorbiaceae. Only three clades, including the ‘Alchornoids’ clade, remain to be examined for the presence of laticifers (Fig. 1). Although our taxon sampling for anatomical analysis is the most phylogenetically comprehensive to date, there is still much to explore in the anatomy of Acalyphoideae, especially considering that it is the most diverse in number of genera (107) of the three major subfamilies of Euphorbiaceae.

Traditionally, before the era of molecular systematics, most taxa of Euphorbiaceae without latex or laticifers were placed in subfamily Acalyphoideae (Webster 1975, 2014; Radcliffe-Smith 2001; Wurdack *et al.* 2005). The characterization of Acalyphoideae as a non-laticiferous lineage was supported by the lack of records in the subfamily, with the exception of a few dubious or poorly documented cases (compiled in Rudall 1987). In her work, Rudall studied 26 genera of Euphorbiaceae s.l. (including taxa from the now-recognized families Peraceae and Phyllanthaceae) and reported the absence of laticifers in Acalyphoideae, based on the anatomical examination of species from seven genera (*Acalypha*, *Dalechampia*, *Mallotus*, *Mareya*, *Mercurialis*, *Plukenetia*, and *Ricinus)* (Rudall 1987, her table 2). However, some of these genera had previous reports for the presence of laticifers or laticifer-like structures (e.g. Pax 1884; Solereder 1908; Metcalfe and Chalk 1950), but none of them could be confirmed by Rudall (1987). Subsequent anatomical studies reporting laticifers in Acalyphoideae are limited to a couple of cases such as the presence of non-articulated laticifers in the wood of *Dalechampia dioscoreifolia* Poepp., the observation of a single laticifer in a fragment of pith tissue of *Acalypha stachyura* Pax (Hayden and Hayden 2000), report of laticifers in *Mallotus glomerulatus* Welzen (van Welzen *et al.* 2004) and in four species of *Macaranga* (Norfaizal *et al.* 2012). However, the last two studies did not provide strong evidence in the form of images from anatomical sections showing laticifers. We argue that due to this paucity of data or poor evidence, the use of the absence of laticifers as a diagnostic feature of Acalyphoideae has prevailed in the botanical literature (Stevens 2001 onward; Wurdack *et al.* 2005; Webster 2014; Pace *et al.* 2019; van Welzen 2020; Levin 2024).

Obtaining solid anatomical evidence (i.e. clear images) for the presence of laticifers appears to be more difficult in Acalyphoideae than in Euphorbioideae and Crotonoideae, the two other main subfamilies of Euphorbiaceae, which in general produce abundant latex easy to observe in the field. Our study demonstrates that the lack of evident exudation in species of Acalyphoideae does not necessarily imply the absence of laticifers. Low latex content or exudation from laticifers, along with the fact that latex can be lost during sample preparation, can hinder the observation of laticifers in anatomical studies (i.e. the cells must have content for the staining process to work). Interestingly, our results show that samples from the petiole appear to be more suitable, or at least less challenging, than those from the leaf blade for assessing the presence of laticifers in low-latex-producing species within Acalyphoideae. In contrast, anatomical studies of *Croton* species without obvious latex exudates in the field have been able to observe laticifers in the leaf blade (Vitarelli *et al.* 2015, 2021; Martín-Muñoz *et al.* 2024). Our results have important methodological implications for future anatomical studies in Acalyphoideae, as there is much to explore in this subfamily, including the three clades not represented in our sampling, but also additional species from the sampled clades and genera.

Similar to our case, only recently detailed anatomical analyses brought to light the few records of laticifers known so far in five genera of Malpighiaceae (*Galphimia*, *Lophanthera*, *Spachea*, *Stigmaphyllon*, *Tetrapterys*) (Anderson 1981, 2001; Vega *et al.* 2002; Pace *et al.* 2019). This highlights the fact that our knowledge about the distribution of laticifers across the tree of life of vascular plants is still incomplete. These knowledge gaps may introduce biases when testing hypotheses in evolutionary biology, such as the escape-and-radiate coevolution hypothesis, i.e. the link between lineage diversification and the evolution of laticifers and ducts in plants (Ehrlich and Raven 1964; Thompson 1989). The fact that Foisy *et al.* (2019) found poor support for this hypothesis when conducting a meta-analytic approach across vascular plants (345 families and 986 genera) could be due in part to this incomplete anatomical knowledge regarding laticifers across many plant lineages.

According to our results, articulated laticifers are probably widespread in the subfamily Acalyphoideae as they were observed in several of the analysed genera including *Acalypha*, *Bia*, and *Dalechampia*. This type of laticifers is also common in the subfamily Crotonoideae with several reports in several genera such as *Astraea*, *Croton*, and *Manihot* (Rudall 1994; Vitarelli *et al.* 2015; Feio *et al.* 2016, 2018). On the other hand, the lack of non-articulated laticifers in our samples should be further confirmed in the same (for further confirmation) and different species of the study genera as well as in other genera of Acalyphoideae. The type of laticifer (articulated vs. non-articulated) has important implications for latex harvesting for commercial purposes and for the conservation of the exploited species. For example, species such as *H. brasiliensis* can be tapped periodically because its laticifers are articulated (Scott 1885; Rudall 1987; Herlinawarti *et al.* 2022), whereas species of *Croton* Dragon’s blood trees, which have non-articulated laticifers (Feio *et al.* 2018), cannot stand periodical tapping without damaging the tree and limiting significantly latex harvesting (Meza 1999; King *et al.* 2020). Additional analyses are required to understand the development of laticifers, because they could be articulated at the beginning of their development and subsequently lose the transversal walls during the process, resulting in non-articulated laticifers when fully differentiated (Demarco *et al.* 2006).

Incorrect assessment of the presence of internal secretory structures such as laticifers and ducts can occur if they are based only on direct observation of the secretion in the field since the resin (stored in ducts) can resemble latex in colour and texture. Resin is produced by the secretory epithelial cells of the ducts, and released from the cytoplasm into the intercellular space (lumen of ducts), while the laticifer cells secrete and store the latex inside the same cell (Fahn 1979). For this reason, detailed anatomical analyses are necessary to identify which of the two structures (laticifer or duct) is responsible for the secretion. In addition, in rare cases, latex can also be synthesized by other cells called unspecialized parenchyma cells, which have been reported in some Euphorbiaceae and may be present independently or along with laticifers (Rudall 1987; Farías *et al.* 2009; Vitarelli *et al.* 2015). See Farias *et al.* (2009) for a detailed review and discussion about these unspecialized latex-producing cells.

## Conclusions

Here we provide the strongest evidence to date that laticifers are likely common in the subfamily Acalyphoideae, being present in representative taxa belonging to six of its nine major clades. This finding refutes the use of the absence of laticifers as a diagnostic feature for Acalyphoideae and expands the application of the presence of laticifers to the entire family Euphorbiaceae. The limited number of anatomical studies with a broad taxonomic representation of Acalyphoideae has probably been influenced by the low abundance or lack of latex exudation in members of this subfamily in field observations (recorded in herbarium labels). We highlight the utility of herbarium material for the detection of laticifers although fresh material is essential for the detailed characterization of the morphology and chemical content of these secretory structures. Future anatomical surveys of laticifers should use a more comprehensive taxon sampling within Acalyphoideae including more representatives per clade as well as members for the three clades (Alchorneioids, A2, and A5) that could not be sampled in this study.

## Supplementary Material

plaf006_suppl_Supplementary_Appendix1

## Data Availability

The data underlying this study are available in the article (figures, table, supplementary appendix). Detailed voucher information, including locality data, collection, and herbarium numbers, for all the samples analysed is provided in Supplementary Appendix 1. All prepared microscope glass slides are physically maintained as part of the slide collection of the Laboratory of Plant Anatomy of the Federal University of Viçosa, Minas Gerais, Brazil. This slide collection is open for consultation by the scientific community.
