# Supplementary material for: Laticifers are present in Acalyphoideae after all: new insights from leaf anatomy with implications for the systematics and evolution of Euphorbiaceae
Source: AoB Plants. 2025 Feb 12;17(2):plaf006. doi: 10.1093/aobpla/plaf006 (PMC11954595; doi:10.1093/aobpla/plaf006)
Supplement: plaf006_suppl_Supplementary_Appendix1 [file plaf006_suppl_supplementary_appendix1.docx]

**Appendix 1.** List of specimens (n = 72) of 40 the species analyzed in this study, indicating their source from either herbarium or fresh material, including voucher number and locality. The voucher of the fresh field-collected specimens (n = 11) is indicated with an asterisk (*). Herbarium acronyms follow Thiers (2024, continuously updated).

***Acalypha accedens* Müll.Arg.**, Simao-Bianchini, R. 565 (SP279979), Cachoeira Paulista, São Paulo, São Paulo, Brazil. ***Acalypha accedens* Müll.Arg.**, Sousa, A.A.C. et al. 31 (SP489614), Road to Parnaso, Petrópolis, Rio de Janeiro, Brazil. ***Acalypha accedens* Müll.Arg.**, Lombardi, J.A.; Salino, A. 1751 (BHCB 37233), District of Fabião, Januária, Minas Gerais, Brazil. ****Acalypha accedens*** **Müll.Arg.**, Mouzella, C.P. 04 (VIC 53539), Pico do Olho D’Água, Mairiporã, São Paulo, Brazil. ***Acalypha amblyodonta* Müll.Arg.**, M.G.C. 862 (BHCB 26682), Tourmaline, Minas Gerais, Brazil. ****Acalypha amblyodonta*** **Müll.Arg.**, Mouzella, C.P. 02 (VIC 53538), Dam Paiva Castro, São Paulo, São Paulo, Brazil. ****Acalypha alopecuroidea* Jacq.**, Mouzella, C.P. 06 (VIC 53541), MRS Company, Jundiaí, São Paulo, Brazil. ***Acalypha brasiliensis* Müll.Arg.**, Cordeiro, I. et al. 3587 (SP 488255), Dores de Paraibuna, Santos Dumont, Minas Gerais, Brazil. ***Acalypha brasiliensis*** **Müll.Arg.**, Sousa, A.A.C. et al. 10 (SP 489606), Itatiaia National Park, Itatiaia, Rio de Janeiro, Brazil. ***Acalypha brasiliensis*** **Müll.Arg.**, Sousa, A.A.C. 51 (SP 489606), Highway R354, access to Ilha do Futuro, Rio de Janeiro, Brazil. ***Acalypha brasiliensis* Müll.Arg.**, Simão-Bianchini, R. 565 (SPF99505), Cachoeira Paulista, Sao Paulo, Brazil. ***Acalypha brasiliensis* Müll.Arg.**, Krieger, L. 1048-A (SP75942), Juiz de Fora, Minas Gerais, Brazil. ***Acalypha brasiliensis* Müll.Arg.**, Assis, L.C.S. 420 (SP357837), Mata do Baú, Barroso, Minas Gerais, Brazil. ***Acalypha brasiliensis*** **Müll.Arg.**, Krieger, L. 21215 (ESA 87524), Juiz de Fora, Minas Gerais, Brazil. ****Acalypha brasiliensis*** **Müll.Arg.**, Sousa, A.A.C. 33 (SP489617), Estrada do Bonfim, Petrópolis, Rio de Janeiro, Brazil. ***Acalypha brasiliensis* Müll.Arg.**, Torrend, C. 111 (SP 35517), Bahia, Brazil. ***Acalypha brasiliensis* Müll.Arg.**, Barreto, H.L.M. 7266 (BHCB 6323), Cambuqueira, Minas Gerais, Brazil. ***Acalypha communis*** **Müll.Arg.**, Barreto, H.L.M. 2688 (BHCB63235), Santa Luzia, Minas Gerais, Brazil. ***Acalypha digynostachya*** **Baill.**, Borgo, M. 238 (BHCB 61418), Fênix, Paraná, Brazil. ***Acalypha diversifolia*** **Jacq.**, Schiavini, I. et al. 222 (BHCB 139376), Fazenda da Mata, Araguari, Minas Gerais, Brazil. ***Acalypha diversifolia*** **Jacq.**, Mota, A.L.P. 1981 (VIC 23893), Mata da Volta Grande, Uberlândia, Minas Gerais, Brazil. ***Acalypha gracilis*** **Spreng.**, Hatschbach, G.M.; Graham, S. 44601 (BHCB 76400), Mineiros Highway, Almirante Tamandaré, Paraná, Brazil. ***Acalypha gracilis*** **Spreng.**, Sousa, A.A.C. et al. 12 (SP 489608), Itatiaia National Park, Itatiaia, Rio de Janeiro, Brazil. ****Acalypha herzogiana* Pax & K.Hoffm.**, Mouzella, C.P. 10 (VIC 53545), Recanto das Cigarras, Federal University of Viçosa, Viçosa, Minas Gerais, Brazil. ****Acalypha hispida*** **Burm.f.**, Mouzella, C.P. 12 (VIC VIC053823), Federal University of Viçosa, Viçosa, Minas Gerais, Brazil. ***Acalypha macrostachya*** **Jacq.**, Mexia, Y. 5022 (VIC 856) Viçosa, Minas Gerais, Brazil. ***Acalypha multicaulis*** **Müll.Arg.**, Araújo, F.S. s.n. (EAC 33254), Serra das Almas, Crateús, Ceará, Brazil. ***Acalypha multicaulis*** **Müll.Arg.**, Castro, A.S.F. 1401 (EAC 32472), Itarumã, Ceará, Brazil. ***Acalypha peckoltii* Müll. Arg.**, Simão Bianchini, 1175 (SP 312879), São Paulo, Brazil. ***Acalypha poiretii* Spreng.**, Linhares, K.M.E. 95 (EAC 55830), Serra das Almas, Crateús, Ceará, Brazil. ****Acalypha poiretii* Spreng.**, Sousa, A.A.C; et al. 45 (SP 489665),"Luiz de Queiroz" Higher School of Agriculture. Nursery near the herbarium., Piracicaba, São Paulo, Brazil. ***Acalypha klotzschii* Baill.**, Costa, L.V; Andrade, I.R; Horta, M.B 291 (BHCB 11304), Caratinga Biological Station, Minas Gerais, Brazil. ***Acalypha velamea* Baill.**, Carvalho, R.C.F de 96 (BHCB 9141), Sete Lagoas, Minas Gerais, Brazil. ***Acalypha villosa* Jacq.**, Hattori, E.K.O. et al. 18 (HUU 34451), Galheiro Environmental Station, Perdizes, Minas Gerais, Brazil. ***Acalypha villosa* Jacq.**, Silveira, A.P. 427 (EAC 40795), Guaramiranga, Ceará, Brazil. ***Acalypha villosa* Jacq.**, Fernandes, A. s.n. (EAC 4216), Serra da Ibiapaba Ubajara, Ceará, Brazil . ***Acalypha villosa* Jacq.**, Nunes, E. s.n. (EAC 16112), Serra de Maranguape, Caucaia, Ceará, Brazil. ***Acalypha villosa* Jacq.**, Nunes, E. s.n. (EAC 8563), Serra do Vicente, Ceará, Brazil. ****Acalypha wilkesiana* Müll.Arg.**, Mouzella, C.P. 11 (VIC 53546), Recanto das Cigarras, Federal University of Viçosa, Viçosa, Minas Gerais, Brazil. ***Acalypha wilkesiana* Müll.Arg.,** Krieger 21215 (ESA 87524), Minas Gerais, Brazil. ***Argythamnia fasciculata* (Vahl ex A.Juss.) Müll.Arg.*,*** Melo, E. 5525 (HUEFS 137261), Morro do Chapéu, Bahia, Brazil. ***Argythamnia* sp.,** Melo, A.C. 5780 (HUEFS 137934), Morro do Chapéu, Bahia, Brazil. ****Bia alienata* Didr.**, Mouzella, C.P. 09 (VIC 53544), Federal University of Viçosa, Viçosa, Minas Gerais, Brazil. ***Caryodendron janeirense* Müll.Arg.**, Neto, S.J.S. 1408 (ESA 120112), Petrobrás Reserve, Duque de Caxias, Rio de Janeiro, Brazil. ***Dalechampia adscendens* Müll.Arg.**, Cordeiro, I. 3544 (SPF 225969), Serra da Canastra National Park, São Roque de Minas, Minas Gerais, Brazil. ***Dalechampia ficifolia* Lam.**, Valente, G.E. 1086 (VIC 26804), Reserva Florestal do Paraíso, Viçosa, Minas Gerais, Brazil. ***Dalechampia humilis* Müll.Arg.**, Pscheidt, A.C; Caruzo, M.B.R; Silva, C.V. 103 (SP 442645), Road between mineiros and Chapadão do Céu, Goiás, Brazil. ***Dalechampia pentaphylla* Lam.**, Fontella, J.P. et al. 1030 (VIC 5283), Texeira, Minas Gerais, Brazil. ***Dalechampia* *triphylla* Lam.**, Moura, L.S. 517 (VIC 7817), Federal University of Viçosa, Viçosa, Minas Gerais, Brazil. ****Dalechampia* sp**. **1**, Mouzella, C.P. 07 (VIC 53542), São Paulo Botanical Garden, São Paulo, Brazil. ***Dalechampia* sp**. **2**, Pirani, J.R. 4807 (SPF 146013), Rodovia Paraíso, Mato Grosso do Sul, Brazil. ***Macaranga heudelotii* Baill.**, Pereira, J.A. 2926 (SP 83179), Hydrophilic formations, Portuguese Guinea. ***Mallotus claoxyloides* Müll.Arg.**, Batianofff, G.N. et al. 11701 (SP 246983), Queensland, Australia. ***Mallotus nesophilus* Müll.Arg.,** Blake, S.T. 17077 (SP 226804), Littoral Forest, Northern Territory, Australia. ***Plukenetia brachybotrya* Müll.Arg.,** Silva, J.A.C. da 104 (INPA 111455), Estrada Porto Velho-Cuiabá, Rondônia, Brazil. ***Plukenetia brachybotrya* Müll.Arg.,** Silva, M.C.R. da 28 (INPA 229921), Lago REBIO, Balbina, Presidente Figueiredo, Amazonas, Brazil. ***Plukenetia loretensis* Ule,** Sobral, M. et al. 9951, (SP 444649), Altamina, Para, Brazil. ***Plukenetia loretensis* Ule,** Santos, J.L. dos 918 (INPA 177605), Reserva Florestal Adolfo Ducke, Manaus, Amazonas, Brazil. ***Plukenetia multiglandulosa* Jabl.,** Silva, M.F. da 155 (INPA36048), Estrada Manaus-PortoVelho, Amazonas, Brazil. ***Plukenetia serrata* (Vell.) L.J.Gillespie.,** Rossini, J. et al. 529 (SP 475924), Augusto Ruschi Biological Reserve, Santa Teresa, Espirito Santo, Brazil. ***Plukenetia serrata* (Vell.) L.J.Gillespie.,** Santos, E.B.; Alves, M.C. 172 (SP 262251), Fazenda Piedade, Una, Bahia, Brazil. ***Plukenetia serrata* (Vell.) L.J.Gillespie.,** Amorim, A.M. et al. 3395 (SP 367632), Terreno do Bosa, Santa Teresa, Espirito Santo, Brazil. ***Plukenetia volubilis* L.,** Panizza, S. s.n. (SP 292378), cultivated, Campinas road, São Paulo, Brazil. ***Plukenetia volubilis* L.,** Lima, E de. 96 (INPA 115525), Margem da Br 422, Tucuruí, Pará, Brazil. ****Ricinus communis* L.,** Mouzella, C.P. 08 (VIC53543), Sao Paulo Botanical Garden, São Paulo, São Paulo, Brazil. ***Tragia incana* Klotzsch ex Baill.**, Gallinal, Rosengurtt et. al; P.E. 4537, Arroyo Timote, Uruguay (SP 51683). ***Tragia* sp.,** Gallinal, Rosengurtt, P.E. et. al. 4537 (SP 51683), Arroyo Timote, Uruguay.
